# Targeted Liposomes: A Nonviral Gene Delivery System for Cancer Therapy

**DOI:** 10.3390/pharmaceutics14040821

**Published:** 2022-04-08

**Authors:** Marcela Tavares Luiz, Jessyca Aparecida Paes Dutra, Larissa Bueno Tofani, Jennifer Thayanne Cavalcante de Araújo, Leonardo Delello Di Filippo, Juliana Maldonado Marchetti, Marlus Chorilli

**Affiliations:** 1 School of Pharmaceutical Science of Ribeirao Preto, University of Sao Paulo (USP), Ribeirao Preto 14040-900, Brazil; marcelatavares@usp.br (M.T.L.); jmarchet@usp.br (J.M.M.); 2 School of Pharmaceutical Science, Sao Paulo State University (UNESP), Araraquara 14800-903, Brazil; jessyca.dutra@unesp.br (J.A.P.D.); larissa_lbt@hotmail.com (L.B.T.); jennifer.thayanne@gmail.com (J.T.C.d.A.); leo.delello@hotmail.com (L.D.D.F.)

**Keywords:** active targeting, DNA, lipoplex, nonviral vector, nucleic acid, RNA

## Abstract

Cancer is the second most frequent cause of death worldwide, with 28.4 million new cases expected for 2040. Despite de advances in the treatment, it remains a challenge because of the tumor heterogenicity and the increase in multidrug resistance mechanisms. Thus, gene therapy has been a potential therapeutic approach owing to its ability to introduce, silence, or change the content of the human genetic code for inhibiting tumor progression, angiogenesis, and metastasis. For the proper delivery of genes to tumor cells, it requires the use of gene vectors for protecting the therapeutic gene and transporting it into cells. Among these vectors, liposomes have been the nonviral vector most used because of their low immunogenicity and low toxicity. Furthermore, this nanosystem can have its surface modified with ligands (e.g., antibodies, peptides, aptamers, folic acid, carbohydrates, and others) that can be recognized with high specificity and affinity by receptor overexpressed in tumor cells, increasing the selective delivery of genes to tumors. In this context, the present review address and discuss the main targeting ligands used to functionalize liposomes for improving gene delivery with potential application in cancer treatment.

## 1. Introduction

Cancer is the medical term used to describe a group of heterogeneous diseases with increasing incidence and representing a global burden on health [1]. Cancer is the second most frequent cause of death in the world, behind cardiovascular diseases, with an estimated incidence increase of about 60% in the coming decades. According to GLOBOCAN 2020 of the International Agency for Research on Cancer (IARC), 28.4 million new cases are expected for 2040, representing a 47% rise compared with 2020. In 2020, IARC estimated 19.3 million new cases and 10 million deaths caused by cancer worldwide. Currently, female breast cancer is the most commonly diagnosed type of cancer (2.3 million new cases, 11.7% of total cases), followed by lung (11.4%) and prostate cancer (7.3%). The larger number of new cases in 2020 occurred in Asia (58.3% of total cases worldwide), followed by Europe (22.8%) and the Americas (20.9%) [2]. The current understanding of the hallmarks of cancer attribute the multistep development of these diseases to six intrinsic biological characteristics of tumor cells, such as: (1) sustained proliferative signaling; (2) increased angiogenesis; (3) resistance to cell death; (4) evading growth factors; (5) replicative immortality: and (6) active invasiveness and metastasis [3].

Global efforts have been focusing on understanding the molecular mechanisms of cancer and discovering new therapeutic targets. Despite many advances in finding more efficient alternatives to treating cancer and patient care, there are still many aspects that need to be improved to make anticancer therapy efficient [4]. Currently, the main problem in cancer therapy is the deleterious adverse effects caused by chemotherapeutic agents, the heterogeneity of cancer, which presents different genetic characteristics and different responses to treatments, and the presence of multidrug resistance mechanisms. Thus, the main objective of pharmaceutical sciences is to develop drugs that are less toxic and more efficient [5]. 

As a promising alternative for the treatment of cancer, gene therapy stands out. This approach comprises the delivery of exogenous foreign genetic material (in vitro or in vivo) to cells with a certain genetic dysfunction to modify the expression of specific genes as a way to circumvent certain clinical conditions [6]. Thus, a wide range of genetic therapies has been reported in the last decades for cancer treatment. Gene therapy has been used to create cancer vaccines [7], to modulate the immune system to achieve cancer cells elimination [8], and to reprogram cancer cells [9]. Although innovative and efficient, gene therapy can generate significant adverse effects, especially immune response. Because of the biological barriers and the host immune system, the delivery of exogenous genetic material can be a challenge, showing low delivery efficiency [10].

Drug delivery nanosystems are capable of overcoming these issues, potentiating the effect of gene therapy through targeted and selective delivery of genetic material to affected cells. Among them, lipid-based nanosystems are the most used nonviral vector for gene expression and silencing, especially the cationic liposomes [11,12]. In the last decades, the literature reports several targeting ligands (e.g., antibodies, antibodies fragments, folic acid, peptides, aptamers, and carbohydrates) that have been used for promoting active targeting of nanocarriers to tumor cells because of their specific recognition and binding by overexpressed receptors [13,14,15,16]. Thus, substantial progress has been made in the delivery of plasmid DNA, mRNA, microRNA (miRNA), small interfering RNA (siRNA), short hairpin RNA (shRNA), and antisense oligonucleotide therapies for cancer treatment with promising results [17]. In this context, the present review addresses the most recent research articles that employed liposomes as a nonviral vector for cancer gene therapy, discussing the main results obtained using in vitro and in vivo studies.

## 2. Gene Therapy

The American Society of Gene and Cell Therapy defined gene therapy as the “introduction, removal, or change in the content of a person’s genetic code with the goal of treating or curing a disease” [18]. It is a promising therapeutic approach used in the treatment of infectious and genetic disorders [19], which uses different nucleic acids (NAs) such as siRNA, shRNA, miRNA, CRISPR system, and antisense oligonucleotides [20].

Unlike traditional therapies, which can present a short response without continuous dosages, gene therapy presents potential durable effects even after being administered in singles doses. However, when compared with traditional drugs, the high costs of gene therapy can be explained by the specialized manufacturing process, difficulty in clinical trials, and clinical delivery requirements [21].

The first clinical trial using gene therapy was performed in the 1990s on a patient with adenosine deaminase deficiency and severe combined immunodeficiency [22]. However, in 1999, the first case of death using gene therapy was reported in a patient with ornithine transcarbamylase deficiency as a consequence of a strong systemic inflammatory response due to treatment with hematopoietic stem cells modified with retroviral vectors [22]. As a result of this death, the research involved with gene therapy declined until 2010, especially in the United States and Europe. In this period, only four products were approved in Asia for gene therapy, including (1998) Vitravene—cytomegalovirus retinitis to immunosuppressed patients; (2003) Gendicine—head and neck cancer; (2005) Oncorine—nasopharyngeal carcinoma; and (2011) Neovasculgen—peripheral vascular disease and limb ischemia [23].

In the last decades, intense research and preclinical safety studies have been conducted, resulting in successful clinical applications. Since then, several products based on gene therapy have been approved, including those based on RNA interference (RNAi) (Lumasiran, Patisiran, and Givosiran), antisense oligonucleotides (Golodirsen, Mipomersen, Etaplirsen, and Nusinersen), recombinant adeno-associated viral vectors (Onasemnogene abeparvovec, Alipogene tiparvovec, and Voretigene neparvovec-rzyl), and lentiviral-transduced cells (Axicabtagene ciloleucel, Autologous CD34+ cells transduced with a lentiviral vector containing the human ADA gene, Brexucabtagene autoleucel, and Tisagenlecleucel) [18].

It is estimated that more than 2200 clinical trials related to gene therapy have been conducted worldwide since 2015 [24]. The main clinical trials are taking place in the United States (65%), followed by Europe (23.2%) and Asia (6.5%) [25]. Other countries, including Australia, Canada, Russia, the Republic of Korea, and Twain, are responsible for a small percentage of research (7.4%) [26]. The percentage of clinical trials with gene therapy is around 38.3% in phase I, 14.2% in phase II, 4.4.% in phase III, and only 2.2% in phase IV [26]. Among them, clinical trials for cancer treatment have represented 67% of all research, being justified by its increased worldwide incidence and lack of effective therapeutic therapies. Some clinical trials with gene therapy for cancer treatment are listed in Table 1.

Gene therapy can be classified into two principal mechanisms. The first mechanism (also called “traditional gene therapy”) is related to the direct addition of gene copy which encodes a correct product to the cell’s genome. In this mechanism, the gene can be directly integrated into the genome, or it can exist as a free-standing segment of DNA. The second mechanism refers to the edition of genes with clinical potential, involving their modifying, replacement, augmentation, and defect blockage [24,27]. The fi-rst gene-editing therapy was used to prevent HIV infection in T cells in 2010, while the gene modification was observed in the CRISPR system for beta-thalassemia and sickle cell disease [28].

**Figure 1:**
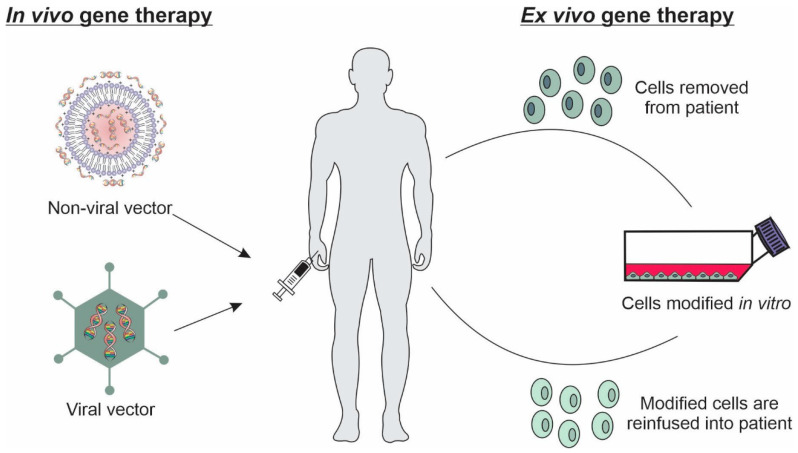
In vivo and ex vivo techniques related to gene delivery to target cells.

Basically, in vivo and ex vivo techniques can be used in gene therapy. In vivo technique is the most used approach in gene therapy, based on the direct introduction of the specific genetic material to target cells or tissues. For this purpose, different physical and chemical methods can be used, such as sonoporation, electroporation, photoporation, gene gun, and delivery systems [26]. In the ex vivo technique, specific cells are removed from the host, cultured, and genetically modified by in vitro transfection, being subsequently retransplanted into the patients (Figure 1) [29].

In addition to in vivo and ex vivo techniques, different considerations are required in gene therapy as the size of the gene to be introduced, stability of the gene expression, target cell type, safety, and efficiency of the gene delivery system [22]. Furthermore, one of the main challenges is to perform a specific targeting of genes into tumor cells, avoiding the normal cells. In this way, gene delivery systems called “vectors” have been used. [30,31].

In gene therapy, the success of the treatment depends on the gene vectors because they are responsible for delivering the therapeutic genes through a process called transfection. Vectors are vehicles capable of protecting the therapeutic gene and transporting it into cells, tissues, and organs. The vector can mediate the delivery of these therapeutic genes because of the size of the NAs, negative charge, and susceptibility to nuclease-mediated degradation that prevent the delivery of the naked NAs. They are mainly categorized as viral and nonviral vectors [32,33,34].

Viral vectors are artificial viruses that contain gene cassettes encoding desirable features in place of the viral genome. In gene therapy, retroviral, adenoviral, adeno-associated virus, herpes viral vector, and vaccine vectors are used as vehicles. Each vector has disadvantages and advantages, and therefore the choice of viral vector depends on the type of cancer, therapeutic strategy, and viral tropism [34]. In general, viral vectors have shown high efficiency in transfection and gene expression through their ability to infect cells productively, such as a wild-type virus [35,36,37,38]. Despite being more efficient at transfection than nonviral vectors, their use has limitations due to immunogenicity and toxicity. Among the problems are viral tropism (e.g., herpes simplex virus has a neuronal tropism), inability to transduce nondividing cells (e.g., retroviruses), low titer production (e.g., adeno-associated viruses), inflammatory potential (e.g., adenoviruses), small capacity of inserted foreign DNA (e.g., retroviruses and adeno-associated viruses), high host immune response (e.g., adeno-associated viruses), and high dose-related toxicity (e.g., herpes simplex virus) [34,35,39]. There is also the phenomenon of insertional mutagenesis, in which ectopic chromosomal integration of viral DNA disrupts tumor suppressor gene expression or activates oncogenes with tumorigenic potential (e.g., lentivirus and cytomegalovirus) [34,37]. Another challenge to using a viral vector is related to the high cost involved in the downstream processing, in which the purification steps require effective and reproducible methods [40]. In addition, viral vectors are not available for codelivery of NAs and drugs. It occurs because transfection efficiency is not target dependent, and the therapeutic molecules available for insertion into the vectors are only NAs [32,36].

The nonviral vector systems are promising for cancer gene therapy, mainly because of their biosafety, low immunogenicity, and low pathogenicity. They have a flexible chemical design that can be modified to achieve physicochemical properties favorable for delivering NAs to target cells. They protect the therapeutic gene, enhance their cellular uptake, and can bind and condense NAs of various sizes (e.g., plasmid DNA and oligonucleotides) because of their greater packing capacity as compared with viral counterparts. Besides favoring the codelivery of NAs and drugs, other advantages include ease of production and the potential for repeat administration [11,32,34,41]. In recent years, different nonviral vectors have been developed with an important role in new therapies, such as liposomes, solid lipid nanoparticles, nanostructured lipid carriers, nanoemulsion, polymeric nanoparticles, dendrimers, gold nanoparticles, and quantum dots [17,34,42].

## 3. Liposomes as a Nonviral Vector

Lipid-based nonviral vectors are a promising alternative to viral vectors for gene delivery, especially liposomal formulations. The production of liposomes is a strategy that facilitates the delivery of therapeutic genes into cells because they are spherical vesicles consisting of one or more phospholipid bilayers, similar to the lipids present in biological membranes. Furthermore, liposomes have low immunogenicity and toxicity due to the modulation of composition, size, and charge. These vesicles can be characterized according to their size (small, large, and giant), charge (cationic, anionic, and neutral), number of lamellae (unilamellar, multilamellar, and multivesicular), surface modification, and lipid composition [11,17,43,44].

**Figure 2:**
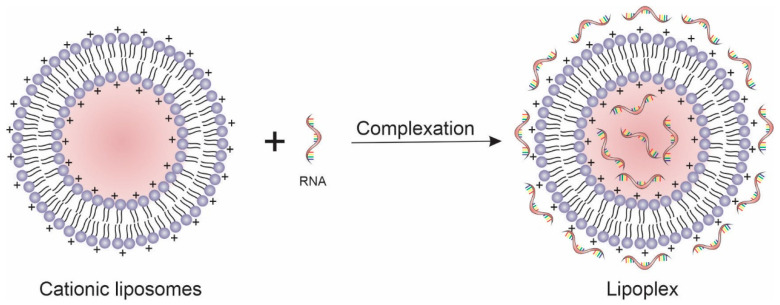
Schematic representation of lipoplex formation.

The lipids used to form liposomes can be cationic, anionic, neutral, or a mixture of them. The lipid used to prepare liposomes can influence the method by which NAs are inserted into the formulation, whicsh can be by complexing the NAs on the surface of the liposomes (liposomes/NAs complex) or encapsulating the NAs within their aqueous core (NAs-encapsulated liposomes) [16,45,46]. The anionic lipids commonly used in the development of liposomes are the phospholipids that can be found naturally in cell membranes, such as phosphatidic acid, phosphatidylglycerol, and phosphatidylserine. These lipids can modulate the physical properties of the membrane, ensuring greater stability by preventing inactivation in the presence of serum. They have more favorable biocompatibility because of their natural presence in biological membranes. Since these lipids have a negative charge, NAs cannot be complexed and should be encapsulated in the liposomes during the vesicles’ formation. The negative charge of these lipids prevents the efficient compacting of the DNA through repulsive electrostatic forces that occur between the phosphate group of the DNA and the anionic head groups of the lipids, decreasing the transfection efficiency. For this reason, cationic lipids are preferable to anionic ones for gene transfer. The complexation between cationic lipids and nucleic acids forms ordered phase structures, also known as lipoplexes (Figure 2) [47,48]. Cationic lipids are amphiphilic molecules structurally similar to natural lipids but positively charged because of the presence of a cationic headgroup [11,32]. NAs can be encapsulated in cationic liposomes through their addition during the liposomes’ formation step (e.g., the inclusion of NAs in the hydration solution in the thin-film hydration method) or by complexation of NAs after liposomes production. In both encapsulation methods, the electrostatic interaction between the negative NAs and cationic lipids is responsible for enhancing the ability of cationic liposomes to carry NAs [8,49,50,51]. Table 2 summarizes some cationic lipids used in gene transfer and their features.

In the lipoplex formation, the electrostatic interaction is the first step of complexation and forms a fast-lasting interaction. This step is associated with the release of 70% DNA counterions and 90% lipid counterions in which structure is stabilized by a neutral interior through the interaction NAs and lipids charges with their counterions inside the complex and a concomitant decrease in hydration. In this step, a higher charge ratio of lipid/NAs forms a faster and more stable complex. The second step is a slower process of irreversible rearrangement and stabilization. In this step, in the first moment, hydrophobic portions of cationic lipids exposed to aqueous media rearrange themselves into unstable conformations. It organizes spontaneously through thermodynamically favorable hydrophobic interactions into a lamellar lipid–DNA complex [52,53].

**Figure 3:**
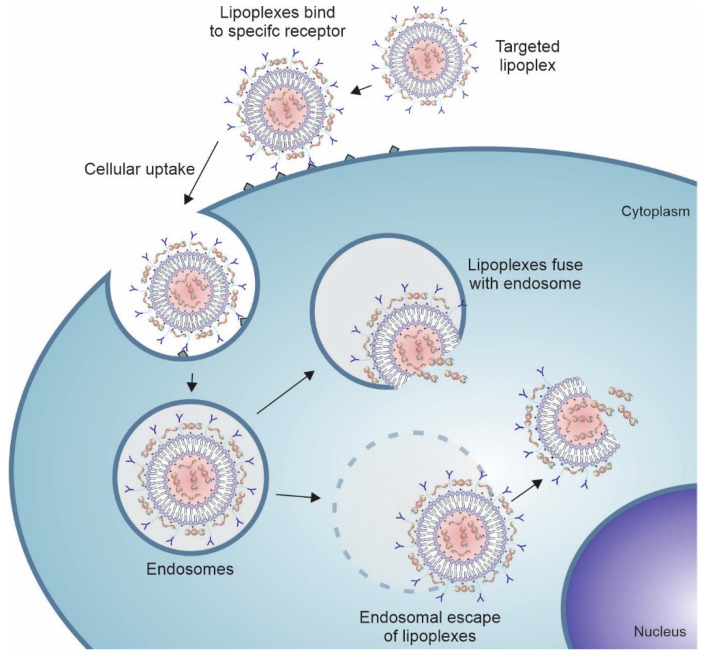
Schematic representation of the cellular uptake of targeted lipoplex and release of NAs into tumor cells. After internalization, lipoplex can fuse with the endosomes or destabilize de endosomes, leading to the release of NAs into the cytoplasm.

Lipoplexes can enhance the delivery of therapeutic genes into cells through mechanisms associated with their chemical structure. Besides the interaction with NAs, the cationic headgroup promotes interaction with the cell membrane through electrostatic interaction with anionic groups of glycoproteins and proteoglycans of the cell membrane, favoring cellular uptake [32,34,41]. Lipoplexes may also increase the transfection efficiency of the NAs in the nucleus through endosomal escape or disruption. The escape from the endosomes occurs by interaction and fusion of the lipoplexes with the endosome membrane, followed by the release of the NAs into the cytosol [11]. The presence of a weakly basic molecule in the headgroup can cause an endosome burst through the proton sponge effect, releasing the NAs into the cytosol of the target cell (Figure 3) [54]. In addition, the inclusion of membrane fusion inducers (e.g., chlorpromazine and procainamide), lysosomotropic agents (e.g., chloroquine), and cell-penetrating peptides with endosomal escape domains in lipoplex can enhance its ability to escape from endosome [55]. Wong-Baeza et al. produced lipoplex containing chlorpromazine, procainamide, chloroquine, and spermidine and observed a significant increase in lipoplex-mediated gene transfection [56]. Lipoplexes are also capable of carrying a large amount of NAs of different molecular weights, such as large plasmid DNAs (Molecular weight < 10 kbase pairs) [57] and messenger RNAs (Mw < 10 kbases) [58], and short sequences (Mw ≈ 15–30 mers) [59].

Lipoplexes share a common structure, including a positively charged polar head (hydrophilic domain) and a hydrophobic tail that are linked through a linker and a backbone domain (e.g., glycerol). The hydrophobic and cationic domains are essential in the composition of lipoplexes [32,34,60].

The polar headgroups of lipids have a large impact on the overall performance of lipoplexes. The size and charge density of this domain influence its interaction with the NAs, the stability of lipoplex, interaction with the cell membrane, the mechanism of endosomal escape, the cytotoxicity, and the compaction of the NAs [61]. Based on the chemical group, this domain can be quaternary ammonium, an amine (primary, secondary, tertiary), an amino acid or peptide, a guanidine, heterocyclic headgroups, and some specific headgroups. Quaternary ammonium is the most frequent group because of its permanent positive charge that provides high solubility in aqueous environments and strong interaction with the NAs [61,62].

The first lipoplex was obtained in 1987 for the introduction of plasmids into cells using the synthetic cationic lipid *N*-[1-(2,3-dioleyloxy)propyl]-*N*,*N*,*N*-trimethylammonium chloride (DOTMA) composed of quaternary ammonium in the hydrophilic domain and a glycerol backbone. It was the first in vivo experiment performed in mice using cationic lipids. The lipoplex was more effective (5 to 100-folds) than either the calcium phosphate or the DEAE-dextran transfection technique [63]. To reduce cytotoxicity and increase transfection efficiency, changes were made to the main DOTMA moieties that produced the first *N*-[1-(2,3-dioleoyloxy)propyl]-*N*,*N*,*N*-trimethylammonium methyl sulfate (DOTAP) [64]. Since then, the quaternary ammonium headgroup has been used in the development of cationic lipids, such as cetyltrimethylammonium bromide (CTAB), dimethyldioctadecylammonium bromide (DDAB) [65], dioctadecanoyl spermines (DOGS), DOTAP [64,65,66], DOTMA [67], and other [61,68].

Searching to evaluate the influence of the hydrophilic domain on the cytotoxicity of cationic lipids, Cui et al. synthesized two cationic lipids with a quaternary ammonium headgroup (CDA14) and a tripeptide headgroup (CDO14) with the same linker bond and hydrophobic domain. The CDA14 (IC_50_ 109.4 μg mL^−1^) was more cytotoxic than CDO14 (IC_50_ 340.5 μg mL^−1^) in the lung cancer cell. Quaternary ammonium headgroup induces more apoptotic cells and reactive oxygen species than peptide headgroup, showing that the toxicity of cationic lipid had a close relationship with their head group structures [68].

Kraz et al. demonstrated the potential of quaternary ammonium-based lipoplexes in inducing a specific immune response for use as a vaccine in cancer immunotherapy. The mRNA-lipoplexes composed of DOTMA/DOPE (1,2-Dioleoyl-sn-glycero-3-phosphatidylethanolamine) or DOTAP/DOPE lipids protected antigen-encoding mRNA from extracellular ribonucleases. Lipoplex (DOTAP/DOPE) efficiently accumulated in the spleen and delivered the mRNA into dendritic cells upon systemic administration. A phase I dose-escalation trial is in progress. First, three melanoma patients were treated with RNA-lipoplexes at a low dose level and were able to produce IFNα and strong antigen-specific T cell responses. Then, a phase I dose-escalation trial produced vaccines with RNA-lipoplexes encoding melanoma-associated malignant antigens, including New York-ESO 1 (NY-ESO-1), tyrosinase, melanoma-associated antigen A3 (MAGE-A3), and transmembrane phosphatase with tensin homology (TPTE) (NCT02410733) [7].

Cationic lipids with tertiary amine-based headgroups, such as DC-Chol (3β-[*N*-(*N*′,*N*′-Dimethylaminoethane)-carbamoyl]cholesterol), are more effective than other amines in the transfection process. As demonstrated by Lin et al., novel lipoplexes based on tertiary amines and DOPE (1:1, *w*/*w*) condensed with siRNA resulted in gene silencing in several cells and normal mice as compared with lipofectamine^®^ (DOSPA: DOPE 1:1, *w*/*w*), a commercially available vector [69], suggesting that tertiary amines being weak bases confer with lipids buffering capacity. This occurs because of the protonation process in an acidic environment of the endosome, favoring the release of NAs inside the cell [11]. 

Heterocyclic head groups such as pyridine, imidazoles, and their derivatives, have been used in the production of lipoplexes because of the chemical feature that can make the amine a stronger or weaker base [70]. Liu et al. synthesized a series of cyclen-based cationic lipids. The authors reported that these lipids were able to originate the proton sponge effect and efficiently release NAs when the pKa of this moiety and the endosome are close, favoring a protonation and endosomal escape [71].

The linker is a moiety between the polar hydrophilic domain and the nonpolar tail(s) of lipids. It plays an important role in the characteristics of cationic lipids, such as stability, biodegradability, transfection efficiency, and cytotoxicity. They are developed with some common chemical groups, such as ether, ester, amide, carbamate, disulfide, urea, acylhydrazone, and phosphate. Other less common types can also be used, such as carnitine, vinyl ether, ketal, glutamic acid, aspartic acid, diamond malonic acid, and dihydroxybenzene [60,72]. At low pH, some linkers (e.g., ortho–ester bonds) are prone to acid hydrolysis, resulting in the weakening of the interactions between cationic lipids and NAs, with consequent release of NAs into the cytosol [73]. To evaluate the influence of linkers on transfection efficiency, Kim et al. synthesized five cationic lipids based on the cholest-5-en-3β-oxyethane-*N*,*N*,*N*-trimethylammonium bromide (Chol-ETA) structure where the cholesterol backbone is linked to the cationic head via various lengths of ether-bound carbon spacer. The transfection efficiency was increased in the order of three < four < two methylene units on their spacer and was decreased by the addition of the isomethyl group to the three-methylene spacer. For the unsaturated spacers to show similar transfection efficiency, the addition of more cationic lipids to the liposome formulation was necessary. Thus, this study suggested that the linkers can influence the transfection efficiency [61].

The hydrophobic domain of cationic lipids can be classified as aliphatic and cyclic (steroid-based) chains [74]. The tail domain of lipoplexes has an influence on the phase transition and fluidity of cationic lipids, as well as an influence on the stability, cytotoxicity, NAs protection, NAs release, endosomal escape, and nuclear penetration of cationic liposomes [74,75]. In general, lipids with two hydrophobic chains (e.g., DOTMA, DOTAP, DOPSA, DORIE, DOGS, and others) are more effective in transfection, probably because of their potential to form stable aggregates in aqueous solutions. However, their transfection results depend on chemical characteristics [71,76]. In contrast, a single tail may present low transfection efficiency depending on the chemical features of cationic lipids. Unsaturated tails can influence transfection, but the major disadvantage of these domains is reduced stability due to susceptibility to oxidation [75,77]. Considering that the chemical structure of the lipid influences transfection efficiency, an alternative would be combining single and double-tailed lipids to evaluate lipoplex uptake and cell transfection efficiency [78,79]. Wang et al. synthesized a series of cyclen-based lipoplexes containing an ester linker and double hydrophobic tails and analogs, including the amide-contained double-tailed lipids, lipids containing ester bonds with the reverse direction, and lipids with a single tail condensed with plasmid DNA. Lipoplexes were less cytotoxic compared with Lipofectamine 2000. Results showed that a double-tailed structure is necessary for efficient transfection of some types of cyclen-based lipids. The length of the hydrophobic chain also largely affects the delivery, and C12 and C14 were found to be the most suitable chain length. The ester-containing lipids were more efficient than their amide bond analogs and exhibited 10-fold higher transfection efficiency than Lipofectamine [80].

Cationic lipids are often mixed with a neutral or auxiliary lipid, also called colipid. The features of cationic lipids and colipids determine the physical parameters of the liposome and can influence the efficiency, colloidal stability, and cytotoxicity of the system [11,60]. Colipids are typically zwitterionic lipids such as DOPE or 1,2-dioleoyl-sn-glycerol-3-phosphatidylcholine (DOPC), or neutral lipids such as cholesterol. In general, zwitterionic lipids promote the conversion of the lamellar phase of the lipoplex into a nonlamellar structure, which improves the transfection efficiency of these complexes [57,60,81].

DOPE is an auxiliary lipid composed of a small hydrophilic phosphoethanolamine domain attached to two bulky unsaturated oleic chains by an ester ligand. It acts as a fusogenic lipid, facilitating the destabilization of the endosomal membrane and a release of NAs into the cell cytosol due to an unstable geometry at acidic pH [81,82]. Cholesterol is a natural steroid found in all animal cell membranes. It is an uncharged molecule and therefore does not interact directly with NAs but supports cationic lipids facilitating interaction with the membrane. It increases the membrane packing of liposomes through interaction with the fatty acid chains assisting in the stability of the liposomal membrane [83]. In a phase I clinical trial, Lu et al. synthesized DOTAP cholesterol condensed to the tumor suppressor gene (TUSC2) expression plasmid (DOTAP:chol-TUSC2) since the TUSC2 gene is often inactivated in lung cancer. It was detected through RT-PCR analysis that eight patients with recurrent or metastatic lung cancer were unresponsive to platinum-based chemotherapy. After treatment with liposomes, seven out of eight patients showed expressions of the TUSC2 plasmid, but not in pretreatment samples and peripheral blood lymphocyte controls. Furthermore, DOTAP:chol-TUSC2 can be safely administered intravenously in lung cancer patients and results in gene uptake by primary and metastatic human tumors (NCT00059605) [84].

Lipoplexes have found considerable utility for in vitro transfection because of their advantages. However, efficient delivery of the genetic material to the nucleus and endosomal lysis are critical parameters that impede the efficiency of gene transfer from lipoplexes. Some liposomal parameters such as size, composition, and positive charge can result in rapid plasma release and immune activation, making application in clinical practice difficult. In addition, various anatomical and cellular barriers (e.g., epithelial and endothelial cell linings and the extracellular matrix surrounding the cells) prevent direct access to the target cells [85]. Despite the limitations, some nonviral lipid vectors show promise in the preclinical stage and are evaluated in the clinic. Table 3 summarizes the currently open clinical trials using lipoplexes in antitumor therapy. An approach used to resolve this challenge is the development of nonviral vectors that are functional or responsive to a biological signal by being able to deliver the content of NAs in a timely and efficient manner.

Another great advantage of using liposomes to deliver NAs is their ability to accumulate in tumors through passive and active targeting. Because of their colloidal properties, they accumulate passively in the tumor tissues through a phenomenon known as the “Enhanced Permeability and Retention effect” (EPR effect), in which there is hypervascularization and reduced lymphatic drainage in the tumor tissue. In contrast, they can be modified by targeting ligands (i.e., proteins, small molecules, peptides, and carbohydrates) that allow the specific targeting of liposomes to cancer cells and tumoral endothelium (Table 4) [44,86,87,88]. This review addresses the most commonly employed target ligands to promote active targeting of lipoplexes in cancer gene therapy and overcome the drawbacks, such as antibodies, peptides, folate, and aptamers.

## 4. Functionalized Liposomes 

### 4.1. Antibodies 

Monoclonal antibodies (mAbs) are relevant components in cancer therapy, targeting tumor cell receptors and simultaneously promoting the induction of a long-term immune response [89,90]. The high specificity and affinity of antibodies have led to the development of treatments with clinical efficacy in cancer therapy, including the approval of drugs for use in cancer treatment [89,91,92]. In 1997, the Food and Drug Administration approved the first mAb for non-Hodgkin’s lymphoma treatment, the rituximab, a chimeric anti-CD20 IgG1 mAb [93]. The multifaceted feature has directed the use of antibodies and their fragments in the engineering of delivery nanosystems at their target site [94].

**Table 4:** List of studies that used liposomes for active targeting nucleic acids to tumoral cells and the effect of functionalization in cancer treatment.

Liposomes’ Composition	Target Ligand	Gene/Drug	Method of Carrying NAs in Liposomes	Particle Size (nm)	Effect of Functionalization	Ref.
DOPC, DOPE, CHOL and DOPE-PEG	Anti-CD44	Triple fusion gene	NAs-encapsulated liposomes	100	Increased in vitro uptake in HepG2 cells, targeted delivery to rat hepatocellular carcinoma, increased transfection efficacy.	[95]
S100-PC, DC-chol, DSPE-PEG, DSPE-PEG_2000_-Mal	OX26 and chlorotoxin	pC27 and pEGFP	NAs-encapsulated liposomes	120	Decrease encapsulation efficiency by about 1.5-fold, increase transfection efficiency, decrease tumor size and increase survival time in mice.	[49]
DMKE, Chol, DSPE-mPEG_2000_, and DSPE-PEG_2000_-Mal	Cetuximab	Vimentin or JAK3	(1) NAs-encapsulated liposomes (2) Liposomes/NAs complex	(1) 173.1 (2) 153.1	Increased in vitro cell binding to EGFR, increased transfection efficiency in vitro and in vivo, increased antitumor activity in vitro in EGFR-positive cells, specific targeting in vivo, complete regression of tumors without lung metastasis.	[45]
DMKE, Chol, DSPE-mPEG_2000_, and DSPE-PEG_2000_-Mal	Cetuximab	Salmosin or IL12	(1) NAs-encapsulated liposomes (2) Liposomes/NAs complex	(1) 173.1 ± 7.5 (2) 153.1 ± 4.2	Increased in vitro cell binding to EGFR, increased transfection efficiency in vitro, and in vivo increased expression of genes in vivo.	[8]
DC-chol, DOPE, DSPE-PEG_2000_-Mal, and DSPE-mPEG_2000_	Anti-HER2 Fab’	Anti-RhoA siRNA	Liposomes/NAs complex	130 to 150	Increased in vitro cell binding to HER1, increased transfection efficiency in vitro, increased RhoA gene silencing efficiency.	[96]
DOPC, DLPC, PEG and PEI	RBDV-IgG1 Fc	pRBDV gene	Liposomes/NAs complex	527.5 ± 83.4	Increased specific delivery in vitro and in vivo, increased transfection and expression of recombinant RBDV protein in 48 h for 7 days in vivo, activation of antibody-dependent cellular cytotoxicity and complement-dependent cytotoxicity, inhibition of tumor growth, and increased survival time.	[51]
POPC, DSPE-PEG_2000_-Mal, DSPE-mPEG_2000_, and DDAB	Anti-CD105	Endostatin gene	Liposomes/NAs complex	122 ± 11	Increased recognition and internalization by the endothelial cell nucleus, increased transfection efficiency, decreased toxicity and tumor size in vivo, decreased targeting of the mononuclear phagocytic system to organs.	[97]
HSPC, DDAB, chol, DSPE-mPEG_2000_	ENG-scFv and ENG-mAb	Porcine α1,3GT gene	Liposomes/NAs complex	103.12 ± 1.5 (scFv) 107.58 ± 2.1 (mAb)	Increased endosomal escape, increased cellular uptake through clathrin-mediated endocytosis in vitro e in vivo, increased induction of hyperacute rejection, increase in anti-αGal antibodies, tumor growth inhibited, and decreased toxicity in vivo.	[98]
DOTAP, DOPE, DSPE-PEG_2000_, l-α-PC, and cholesterol	TAT peptide and trastuzumab	siRNA against the MDR1 gene	NAs-encapsulated liposomes	196.41 ± 0.39	Dual modification of liposomes with TAT peptide and trastuzumab enhanced the cellular transfection of siRNA.	[46]
DOTAP, iRGD-PEG_2000_-DSPE, mPEG_2000_-Chol, and cholesterol	iRGD peptide	Pigment epithelium-derived factor-DNA (PEDF-DNA)	Liposomes/NAs complex	240	iRGD-modified liposomes enhanced the cellular of PEDF-DNA, which suppressed angiogenesis and enhanced apoptosis.	[9]
DPPC, DSPE-PEG-Mal/OCH_3_, DOTAP, and cholesterol	iRGD peptide	Antisense oligonucleotides against androgen receptor (AR-ASO)	NAs-encapsulated liposomes	150 ± 36	iRGD-liposomes increase AR-ASO transfection in the tumor tissue and reduce androgen receptor expression.	[99]
DOTAP, cholesterol, DSPE-PEG_2000_	iRGD peptide	shRNA against elF3i	Liposomes/NAs complex	100	iRGD-liposomes effectively transfect B16F10 cells. In vivo studies indicated that this formulation downregulated eIF3i expression, inhibiting metastasis and cell proliferation.	[100]
DSPE-PEG_2000_, EPC, and PSH	cRGD peptide	Survivin siRNA	Liposomes/NAs complex	131.87 ± 8.45	cRGD-liposomes showed great gene silencing and antitumor activity both in vitro and in vivo.	[101]
EPC, cholesterol, DSPE-PEG, and DOTAP	GE-11 peptide	HIF1α-siRNA	Liposomes/NAs complex	166.4 ± 1.45	The synergic effect of gemcitabine and HIF1α-siRNA loaded in GE-11-modified liposomes reduced the tumor fourfold more than in the control group.	[102]
EPC, cholesterol, stearamide, and DSPE-PEG_2000_	tLyp-1 peptide	miRNA against Slug gene	Liposomes/NAs complex	120	TLyp-1-modified liposomes enhance the transfection of miRNA in MDA-MB-231 cells and silenced the Slug gene and protein expression in vivo.	[103]
DPPC, cholesterol, DPPE-PEG750, and PEI	PR_b peptide	miRNA 603 (miR-603)	NAs-encapsulated liposomes	141 ± 34	PR_b-modified liposomes enhanced the cellular transfection of miR-603 and the radiation sensitivity of patient-derived glioblastoma stem-like cells.	[16]
DOTAP, cholesterol, DSPE-PEG_2000_, an DOPC	Angiopep-2	GOLPH3-siRNA	Liposomes/NAs complex	88.0	Angiopep-2-modified GOLPH3-siRNa-loaded liposomes were able to accumulate in the brain and inhibit glioma growth.	[104]
Dc-Chol, DOPE, and MAL-PEG-NHS	AS1411 Aptamer	Anti-BRAF siRNA (siBraf)	Liposomes/NAs complex	150	Anti-BRAF siRNA accumulation in melanoma cells, with BRAF gene silencing in vitro and in vivo	[105]
DOPE, sphingomyelin (SM), cholesterol, DSPE-PEG_2000_, Didecyldimethylammonium bromide (DDAB)	AS1411 Aptamer	siRNA PLK1 and paclitaxel	Liposomes/NAs complex	121.27	Reduced polo-like kinase 1 mRNA expression, induced apoptosis, and reduced angiogenesis and systemic toxicity in vivo	[106]
DPPC, cholesterol, mDSPE-PEG_2000_	AS1411 Aptamer	siRNA Notch 1 and protamine	Liposomes/NAs complex	285	Notch 1 gene silencing and potentiation of the anti-proliferative effect	[15]
DOTAP and cholesterol	A10 Aptamer	CRISPR/Cas9	Liposomes/NAs complex	150	Polo-like kinase 1 gene silencing, induction of apoptosis, and tumor reduction in vivo	[107]
DPPC, cholesterol, DSPE-PEG_2000_	Anti-CD44 Aptamer	siRNA and protamine	Liposomes/NAs complex	137	Reduced luciferase activity in vitro and in vivo	[50]
DOTAP and cholesterol	EGFR Aptamer	SATB1 siRNA	Liposomes/NAs complex	161.2	Inhibition of the SATB1 gene in vitro and in vivo, increased cytotoxicity in vitro, and inhibition of choriocarcinoma xenograft tumor in vivo	[14]
HSPC, DOTAP, cholesterol, DSPE-PEG_2000_-COOH	Epithelial cell adhesion molecule (EpCAM) Aptamer	miR-139-5p	Liposomes/NAs complex	150.3	Greater accumulation in tumor tissue and reduction in tumor volume in vivo	[108]
1,26-bis (cholest-5-en-3β-yloxycarbonylamino)-7,11,16,20-tetraazahexacosan tetrahydrochloride, DOPE, and lipoconjugate	Folate	Anti-MDR1 siRNA	Liposomes/NAs complex	60 ± 22	Folate-modified liposomes enhanced the siRNA transfection 3–4-fold in comparison with the unmodified formulation.	[109]
DOPE and lipoconjugate	Folate	Anti-MDR1 siRNA	Liposomes/NAs complex	175.2 ± 22.6	FA-modified liposomes effectively accumulate in tumors with overexpression of folate receptors.	[13]
DOTAP, cholesterol, DSPE-PEG_2000_, and folate-PEG-CHEMS	Folate	Anti-Bmi1 siRNA and ursolic acid	Liposomes/NAs complex	165.1	Folate-modified liposomes codelivering Bmi1 siRNA and ursolic acid demonstrated significant higher cellular uptake and antitumoral effect than the unmodified liposomes	[110]
DOTAP, cholesterol, and mPEG-Chol	Folate	PEDF plasmid	Liposomes/NAs complex	200	Folate-modified PEDF plasmid-loaded liposomes inhibited cell proliferation and induced apoptosis of cervical cancer cells in vivo.	[111]

Legend: Dc-Chol: 3β-[*N*-(*N*′,*N*′-Dimethylaminoethane) carbamoyl] cholesterol, DOPC: 1,2-Dioleoyl-sn-glycero-3-phosphocoline, DOPE: 1,2-dioleoyl-sn-glycero-3-phosphoethanolamine, DOTAP: 1,2-dioleoyl-3-trimethylammonium-propane, DPPC: Dipalmitoyl phosphatidylcholine, DPPE-PEG750: 1,2-dipalmitoyl-sn-glycero-3-phosphoethanolamine-*N*-[methoxy(polyethylene glycol)-750], DSPE-PEG_2000_: 1,2-distearoyl-sn-glycero-3-Phosphoethanolamine polyethyleneglycol-2000, eLF3i: eukaryotic translation initiation factor 3i, ENG-scFv: anti-endoglin single-chain Fv fragments, EPC: phosphatidylcholine, folate-PEG-CHEMS: folate-polyethylene glycol-cholesteryl hemisuccinate, Mal-PEG-NHS: Maleimide poly(ethylene glycol) succinimidyl valerate, mPEG_2000_-Chol: mPEG_2000_-succinyl-cholesterol conjugate, OX26: anti-transferrin receptor antibody, PEI: polyethyleneimine, PSH: reduction-sensitive cationic polymer, RBDV-IgG1 Fc:RBDV and IgG1 Fc recombinant fusion proteins.

**Figure 4:**
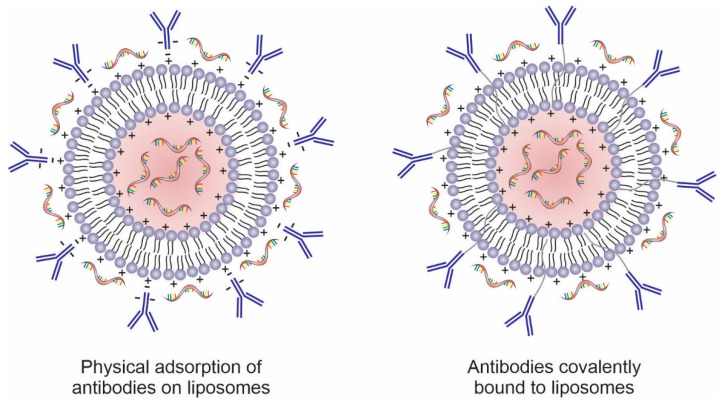
Schematic representation of antibodies physically adsorbed and covalently conjugated to the surface of nucleic acids-loaded liposomes.

Antibodies can be physically or covalently conjugated to the surface of these nanosystems, including liposomes (Figure 4). Surface modification of liposomes with mAbs increases intracellular uptake, via endocytosis, through specific recognition of cell surface proteins or receptors overexpressed on the cancer cell membrane or in the tumor microenvironment [86,112,113]. Several tumor cell surface markers are identified as specific and selective targets for anticancer therapy and present promising results as targeting agents in nanosystems such as CD44 [114,115], CD147 [116], CD133 [117,118], and CD321 [119]. In addition to whole antibodies, their fragments (e.g., ScFv, ds-Fv, ds-ScFv, and sdAb) have also been used for targeting nanosystems to tumor cells. These fragments contain at least one antigen-binding region to maintain active targeting and have the advantage of being less immunogenic and more stable than full antibodies [120].

CDD4 is a multistructural cell surface glycoprotein expressed on a large number of mammalian cell types. It is involved in signaling physiological activities of normal cell survival such as cell proliferation, differentiation, adhesion, cell migration, angiogenesis, cytokine presentation, and growth factors. Different CD44 isoforms (CD44v) functionally distinct from the standard isoform (CD44s) can be formed through alternative splicing, associated with tumor growth and metastasis. In carcinogenesis, activation of CD44 gene transcription occurs in part through the Wnt signaling pathway—a set of signal transduction pathways in which proteins pass signals into a cell through cell surface receptors [116,121,122]. Hyaluronans are the major ligands of CD44, but some isoforms bind to additional ligands modulating angiogenesis, tumor growth, and invasion. For example, the CD44v6 isoform binds to hepatocyte growth factor and vascular endothelial growth factor (VEGF). The CD44v3 isoform can present fibroblast growth factor to its receptor on target cells, while CD44v6 and v10 isoforms increase prostate cancer cell (PC3) migration [123,124,125]. Since CD44 is a multifunctional molecule, anti-CD44 agents have therapeutic potential in various tumor cells.

Wang et al. developed an anti-CD44 antibody-mediated cationic liposome associated with a triple fusion gene containing herpes simplex virus truncated thymidine kinase (HSV-ttk), renilla luciferase, and red fluorescent protein. The authors evaluated the potential of liposomes to induce apoptosis in hepatocellular carcinoma (HepG2) and, at the same time, assessed the tumor progression using an imaging technique. Targeted liposomes (Lipo-CD44-RB) showed high cellular uptake in HepG2 cells after 1 h of incubation, while untargeted liposomes were not internalized even after 12 h. NOD/SCID mice were treated with anti-CD44-targeted triple fusion-liposomes/ganciclovir (Lipo-CD44-TF/GCV) and nontargeted liposomes (Lipo-TF/GCV). The bioluminescence (BLI) signals showed that Lipo-CD44-TF could specifically target the tumor by recognizing the CD44 antigen, while Lipo-TF/GCV does not target cancer cells. The efficacy of liposomes was evaluated through an in vivo bioluminescence assay, in which the Lipo-CD44-TF/GVC group reduced the tumor growth when compared with Lipo-TF/GVC and the control. The results showed that Lipo-CD44-TF could deliver triple fusion specifically to the tumor but not completely inhibit its growth. This can be explained by the occurrence of GCV-resistant transduced cells due to deletion within HSV-tkk since HSV-tkk, in combination with ganciclovir, turns it into a toxic agent that induces cell death [95].

The transferrin receptor is a membrane glycoprotein encoded by the transferrin receptor gene (TFRC). It is responsible for the internalization of iron-associated transferrin protein via clathrin-mediated endocytosis [126]. Transferrin receptor is often overexpressed in cancer cells (e.g., brain, liver, breast, lung, and colon) since iron is associated with the proliferation and survival of tumor cells [127,128]. Thus, the functionalization of liposomes with transferrin can enhance the transfection of genes in different cancer cells, including brain tumors, squamous cell carcinoma [129], hepatocarcinoma, fibrosarcoma, leukemia, cervical cancer [130], and others [131,132]. Transferrin receptor functionalization is being used to enhance liposomes delivery by antitransferrin receptor antibodies such as OX26 and RI7217 [133,134].

To transport plasmid DNA across the blood–brain barrier (BBB) and target glioma, Yue et al. developed immunoliposomes modified with OX26 antibody and chlorotoxin peptide-loaded with plasmid IRES2-EGFP/hTERTC27 (OX26/CTX-PL/pC27). The cytotoxicity of OX26/CTX-PL/pC27 in glioma cells (C6) was approximately 1.4-fold greater when compared with unmodified liposomes (PL/pC27) but was not significantly different from the liposomes modified with only chlorotoxin peptide (OX26-PL/pC27). No difference in transfection efficiency was demonstrated in HEK293T cells for PL/pC27, OX26-PL/pC27, and OX26/CTX-PL/pC27. However, OX26/CTX-PL/pC27 increased the gene transfection in C6 and F98 cells in comparison with PL/pC27, which indicated the selective delivery of liposomes to C6 cells that overexpress transferrin receptor and MMP-2 (receptor for chlorotoxin peptide). The OX26/CTX-PL/pC27 was able to cross the in vitro BBB model and decrease the cells viability of C6 cells to 46.0%, being more cytotoxic than unmodified liposomes. Dual-targeting therapeutic effects were confirmed with decreased tumor volumes (18.81 mm^3^) and increased mean survival time (46 days) in C6 glioma-bearing mice [49].

Several growth factor receptors, including EGFR/HER1, HER2, and FGFR, are known to be overexpressed on the surface of various cancer cells. Epidermal growth factor receptor (EGFR) is one of the most potent oncogenes usually overexpressed in cancers. Signaling via epidermal growth factor (EGF) leads to tumor cell proliferation, migration, and invasion by causing dimerization of EGFR, a receptor tyrosine kinase. Anti-EGFR mAb (e.g., Cetuximab and Panitumumab) are conjugated in liposomal formulations to induce apoptosis in tumor cells by blocking ligand binding and receptor dimerization [135,136]. Furthermore, EGRF regulates the subcellular distribution of the transferrin receptor through its tyrosine kinase activity. Thus, its inactivation reduces transferrin receptor expression by suppressing cellular iron import and delaying tumor progression [128].

Kim et al. functionalized negative liposomes, negative virosomes, lipoplex, and cationic virosomes (viroplex) with anti-EGFR (cetuximab) for targeting two siRNA for cancer cells expressing EGFR. The authors used siRNA against Janus kinases, a protein essential for cell proliferation and survival, and vimentin, a structural protein related to tumor adhesion, migration, and survival. Viroplex and virosomes were produced with the insertion of Sendai virus F/HN protein during liposomes production. The immunolipoplex showed higher cellular bind to SKOV-3 cells (EGFR-positive) compared with immunoliposomes and nonfunctionalized liposomes, whereas they showed less binding to EGFR-negative B16BL6 cells. A pretreatment with free cetuximab reduced the uptake of immunolipoplex by cancer cells in cytometry analysis, evidencing that the targeting ability of immunolipoplex is due to the specific binding between cetuximab and EGFRs overexpressed on the cancer cell surface. The targeted lipoplexes showed higher transfection efficiency in SKOV-3 cells, whereas nontarget lipoplexes showed high transfection in B16BL6 cells. By fluorescence microscopy analysis, BALB/c nude mice with SKOV-3 tumors treated with rhodamine-labeled anti-EGFR formulations showed increased fluorescence intensity in the tumor region, showing specific targeting. Furthermore, the combination of doxorubicin and immunolipoplex/immunoviroplex containing both siRNA had a remarkable antitumoral effect, with a significantly higher reduction in tumor volume [45].

Previously, Kim et al. functionalized liposomes (neutral surface charge) and lipoplexes with anti-EGFR to loaded salmosin and interleukin 12 (IL12) genes for the treatment of human ovarian adenocarcinoma (SKOV-3), lung adenocarcinoma (A549), and breast carcinoma (MCF-7), and mouse melanoma (B16BL6). IL-12 and salmosin have an antiangiogenic function. In addition, IL12 activates NK cells and cytotoxic T cells. Targeted formulations showed greater cellular binding affinity to A549 and SKOV-3 cells (EGFR-positive) than MCF-7 and B16NL6 cells (EGFR negative), indicating the selectivity of both formulations for EGFR. Furthermore, the untargeted formulation showed a high binding affinity to all cell lines. According to the results of expression luciferase, EGFR-positive cells were effectively transfected by anti-EGFR immunolipoplexes, but EGFR-negative cells were not, being immunolipoplexes more efficient than immunoliposomes. Histological analysis of SKOV-3-xenografted nude mice treated with the formulations showed that the immunolipoplexes containing pIL12/pSal reduced the tumor volume and lung metastasis [8].

Similar to EGFR, overexpression of HER2 in tumors, mainly human breast carcinoma, is associated with more aggressive disease and poor prognosis. It is a receptor tyrosine kinase that has no specific ligand, being activated by heterodimerization with other growth factor receptors. HER2 is associated with resistance to endocrine therapy, which causes the hormone receptor and receptor tyrosine kinase pathways to stimulate tumor cell proliferation. The overexpression of this receptor in tumors allows its use as a target for nanosystems-loading genes [137,138].

Gao et al. developed PEGylated immunoliposomes with anti-HER2 Fab’ (PIL) by extrusion and a lyophilized PIL (LPIL) for anti-RhoA siRNA delivery to HER2-overexpressing cancer cells. Among a series of LPIL with different degrees of PEGylation, LPIL containing 2.5% PEG (2.5% PEG LPIL) showed better gene transfection efficiency by showing approximately 2.5 times more fluorescence in SK-BR3 (HER2-positive) cells than the other PEG concentrations. PIL showed approximately 7 times lower fluorescence when analyzing HER1 expression in SK-BR3 cells by flow cytometry assay, showing that lyophilized LPIL has better gene silencing efficiency when compared with PLI. Confocal microscopy studies demonstrated that 2.5% PEG LPIL is specifically targeted and internalized in SK-BR3 cells. 2.5% PEG LPIL with anti-RhoA siRNA showed approximately fivefold decreased proliferative capacity in SK-BR3 cells compared with 2.5% PEG LPL and untargeted formulations with anti-RhoA siRNA. The results demonstrated the potential of the functionalization of liposomes with anti-HER2 Fab’ for promoting specific siRNA transfection and gene silencing [96].

Targeting nanosystems to the tumor microenvironment is another strategy that offers benefits to anticancer therapy. The tumor microenvironment contains factors involved in inhibition of the antitumor immune response, tumor cell growth and induction of pro-tumor angiogenesis [12,51,139,140]. Ho et al. developed cationic immunoliposomes complexed with an antiangiogenic RBDV-IgGG1 Fc fusion protein (LPPC). A plasmid (pRBDV) was complexed to cationic liposomes to evaluate interference with the VEGF-VEGFR axis signaling pathway, which promotes angiogenesis. LPPC complex exhibits higher fluorescence intensity in VEGFR-positive cells (B16-F10 cells) than VEGFR-negative cells (BALB/3T3 cells), showing that the LPPC specifically targeted VEGF receptors. LPPC complex increased transfection by 3.5-fold in B16-F10 cells compared with the untargeted liposome in flow cytometry analysis. VEGFR-positive transfectants expressed RBDV after transfection (4 µg·mL^−1^ protein in 48 h), which was shown to be bioactive through antibody-dependent cellular cytotoxicity and complement-dependent cytotoxicity. Imaging in male C57BL/6 mice (bioluminescence assay) showed that LPPC complexes could reach B16-F10 tumors in 72 h and not accumulate in other organs. After LPPC/pRBDV/RBDV treatment, the mice expressed the recombinant proteins for 7 days, inhibited tumor growth, and improved survival time (100% in 35 days) [51].

Tumor-associated endothelial cells proliferate rapidly because of the essential role of angiogenesis in tumor development and dissemination. Endoglin is an endothelial cell membrane glycoprotein (also known as CD105) that actively participates in blood vessel development and is another promising target in cancer therapy [141].

Zhuo et al. developed cationic immunoliposomes (immunoliposomes/pcDNA) modified with anti-CD105 antibody and loaded with the pcDNA3.1-CSF1-endostatin gene for tumor-specific imaging and antiangiogenic activity. Cellular uptake of immunoliposomes/pcDNA was determined in CD105 positive endothelial cells using calcein as a dry. immunoliposomes/pcDNA was recognized and internalized by endothelial cells with partial localization in the nucleus, indicating active cell-to-cell transport by fluorescence-activated cell-sorting analysis in microvascular endothelial cells derived from primary tumors (CD105 positive). The gene transfection efficiency of immunoliposomes/pcDNA increased by 35% when compared with the unmodified cationic liposomes (liposomes/pcDNA). No toxic effects were observed in Kunming mice 5 and 17 days after injection of liposomes/pcDNA or immunoliposomes/pcDNA. Nude mice bearing MDA-MB-231-Luc xenografts showed a gradual increase in fluorescence up to 12 h that persisted for more than 72 h while the fluorescent signal decreased to near baseline levels in organs of the mononuclear phagocytic system on multispectral fluorescence, luminescence, and digital X-ray capacity analysis in a single system. Tumor size decreased 2.6-fold after 42 days following immunoliposomes/pcDNA injection (229.75 ± 53.91 mm^3^) compared with PBS (611.85 ± 71.96 mm^3^) [97].

To develop immune stimulation against tumor cells, Huang et al. relied on the specificity of Galα1-3Galβ1-4GlcNAc-R (αGal) carbohydrate epitope synthesis by endoglin (α1,3-galactosyltransferase—α1,3GT) to express αGal on the surface of cells in the tumor microenvironment. The authors produced PEGylated cationic immunoliposomes loaded with α1,3GT (LPs/α1,3GT) and complexed to anti-endoglin single-chain Fv fragments (ENG-scFv-LPs/α1,3GT) or anti-endoglin monoclonal antibody (ENG-mAb-LPs/α1,3GT). The hENG-HEK 293 cells were treated with the LPs/α1,3GT, ENG-mAb-LPs/α1,3GT, and ENG-scFv-LPs/α1,3GT for 8 h, and the cellular localization α1,3GT was observed by confocal microscopy. The average fluorescence intensity was highest upon incubation with ENG-scFv-LPs/α1,3GT, being colocalized with endosomes stained with LysoTracker Red™. After 6 h, almost all green fluorescence was separated from the LysoTracker signal in the ENG-scFv-LPs/α1,3GT group, demonstrating endosomal escape. The synthesis of αGal catalyzed by α1,3GT in hENG and TnECs cells was evaluated after incubation for 72 h. Cells treated with ENG-scFv-LPs/α1,3GT showed stronger red fluorescence in the cytoplasm than cells treated with ENG-mAb-LPs/α1,3GT, showing that there was greater induction of hyperacute rejection. The in vivo biodistribution in A549 tumor in NOD/SCID mouse was 3.7-fold higher than in the liver 72 h after the injection of ENG-scFv-LPs. The antitumor efficacy of ENG-scFv-LPs/α1,3GT was investigated and shown to be positive for anti-αGal antibodies. The levels of IL-12p70 and INF-γ (activation cytokines of NK cells, CD8+ T cells, and macrophages) in mice treated with ENG-scFv-LPs/α1,3GT were significantly higher than those in ENG-mAb-LPs, while the levels of IL-10 and TGF-β (immunosuppressive cytokines) decreased, showing an antitumor immune response. After treatment with ENG-scFv-LPs/α1,3GT once a week for five weeks, tumor growth was inhibited, with no noticeable change in body weight, and survival increased. Blood circulation time and tumor accumulation of liposomes, as well as site-specific delivery of α1,3GT, were increased, resulting in optimal therapeutic efficacy [98].

Monoclonal antibodies are important in clinical practice against cancer, but therapeutic resistance is still a challenge. The combination of the specificity of mAbs with the versatility of liposomal systems has demonstrated that there are promising alternatives to maximize the benefit of the use of liposomes and mAbs in cancer gene therapy by increasing the functionality of liposomes and therapeutic fronts in cancer therapy. As mentioned above, immunoliposomes possess the ability to activate antibody-dependent cellular cytotoxicity while directionally delivering one or more nucleic acids to the target cell, decreasing toxic effects, increasing efficacy, and creating an antitumor immune response.

### 4.2. Peptides

Peptides are short amino acid chains, often less than 40 units, that have their origin in natural or synthetic sources. Over the past few decades, the functionalization of liposomes with peptides has received attention because of their ability to improve the transport of nanoparticles through biological membranes and enhance cellular internalization [142]. In comparison with antibodies, peptides have a lower production cost, higher penetration ability, and less immunogenicity. Despite their lower binding affinity (1~10%) when compared with antibodies, their smaller molecular weight enables multiple copies to incorporate into the liposomes’ surface area, which increases their binding affinity [143]. In a general way, peptides can be classified into cell-penetrating peptides (CPP) and tumor targeting/homing peptides. CPPs are cationic, amphipathic, or hydrophobic peptides (e.g., transactivator of transcription (TAT) peptide, penetratin, rabies virus-derived peptide, chimeric rabies virus glycoprotein fragment, and octaarginine peptide) that have been used to increase the cellular uptake of liposomes for delivering therapeutic agents. The cellular uptake mechanism of CPPs is still understood. It is not yet known if the cellular uptake o CCPs are mediated or not by specific cellular receptors [144,145,146,147]. By contrast, tumor-targeting/homing peptides (e.g., RGD, angiopep-2, Lyp-1, and GE11 peptides) can be recognized by specific receptors in the tumor cells or the tumor microenvironment, being able to differentiate tumor and normal cells [145].

Dehkordi et al. fabricated a liposomal formulation coated with TAT and trastuzumab for improving the intracellular transport of siRNA against multidrug resistance 1 (MDR1) for resensitizing cells to daunorubicin drug. The authors combined the ability of TAT to improve the cellular uptake and lysosomal scape of the liposomes with the active targeting promoted by trastuzumab via HER-2 receptors overexpressed in breast cancer cells. The in vitro results demonstrated that TAT-modified liposomes enhanced the siRNA delivery in MDA-MB-231RDB (daunorubicin resistant) and EPG85.257EDB cell lines in comparison with the unmodified formulation. Furthermore, the association of TAT and trastuzumab potentialized the siRNA uptake. This double surface modification exhibited a 128-fold higher MDR1 silencing effect and protein downregulation around 50 folds, increasing the chemosensitivity to daunorubicin 4.73 times more than the treatment with the free drug without the explosion of TAT-trastuzumab-modified liposomes. In addition, cytotoxicity of daunorubicin in the presence of TAT-modified liposomes (IC_50_ = 14.48 nM) and TAT-trastuzumab-modified liposomes (IC_50_ = 14.28 nM) showed lower IC_50_ than unmodified liposomes (IC_50_ = 30.59 nM) and free drug (IC_50_ = 65.72 nM) in MDA-MB-231RDB. These results indicated the potential of the association of TAT peptide, a CCP, and trastuzumab for improving the cellular uptake of siRNA-loaded liposomes [46].

Fisher et al. have investigated the potential of CPP-loaded noncationic liposomes to deliver siRNA for medical applications since cationic lipids can induce cytotoxicity and immunogenicity in vivo. The authors used octaarginine peptide, a CPP, for improving the siRNA transfection capacity and maximizing the siRNA retention and encapsulation efficiency. For this purpose, the incorporation of octaarginine peptide was performed using modifications via preinsertion, postinsertion, and postconjugation. The authors identified that the pre-insertion technique was the most efficient to produce liposomes with high siRNA encapsulation efficiency (~55%). In addition, octaarginine-loaded liposomes produced by this method showed higher transfection capacity than unmodified liposomes. This study demonstrated the potential of the CPP to enhance the incorporation of siRNA in liposomes and improve the transfection ability, which plays an important role in medical applications [147]. 

Among the homing peptides, linear and cyclic RGD have been the most used to functionalize nanosystems. The linear RGD can bind to multiple integrins, including the α_V_β_3_ integrin, which is overexpressed on the endothelial cells of tumor neovasculature and the tumoral cells. Cyclic RGD peptides (e.g., cRGD and iRGD) can bind more selectively and strongly to integrins than linear RGD. In addition, iRGD binds to neuropilin-1, a receptor also overexpressed on tumor cells [9,148].

Wonder et al. proposed a guideline on how to effectively produce RGD peptide-loaded cationic liposomes to specific target DNA of tumoral cells. For this purpose, they used linear RGD, cRGD, and iRGD peptides. The authors identified the influence of peptide coverage percentage (1, 2.5, 5, and 10 mol%) in the cellular binding and internalization using PC-3 and M-21 cells. The optimal peptide coverage was different for both cells lines; liposomes with 5 mol% peptides had the highest internalization in M-21 cells, while 2.5 mol% was the best for PC-3 cells. The full peptide coverage (10 mol%) results in less internalization in both cell lines because of the lateral steric hindrance. After the optimization of RGD-modified liposomes, the authors performed an in vivo study and demonstrated higher penetration of DNA-loaded liposomes modified with cRGD and iRGD peptides in the tumor tissue. The result demonstrated the ability of cyclic RGD to target tumor cells and the importance of the percentage of peptide coverage on cell internalization [149]. 

Bao et al. developed iRGD-modified cationic liposomes to enhance the intratumoral accumulation of pigment epithelium-derived factor-DNA (PEDF-DNA) in colorectal cancer cells. The PEDF protein can inhibit tumor angiogenesis, tumor cell migration, and induction of apoptosis. Thus, iRGD-liposomes can improve PEDF internalization in tumoral cells and inhibit cancer cell growth and metastasis. The in vitro studies indicated that iRGD-modified liposomes increased the uptake of PEDF-DNA by colon carcinoma cells (CT26) compared with unmodified liposomes. The expression of PEDF-DNA was further investigated in vitro and in vivo by Western blot assay, which indicated that PEDF protein expression was significantly enhanced in iRGD-liposomes. In addition, iRGD-liposomes suppressed angiogenesis, reduced cell proliferation, and induced apoptosis. Furthermore, the developed formulation was able to reduce lung metastasis and prolong the survival time in a mouse model of metastatic colorectal cancer, indicating that the active targeting promoted by the iRGD peptide was efficient to deliver the PEDF-DNA-loaded in liposomes [9]. 

Another study performed by Guan et al. has also investigated the potential of iRGD peptide-modified liposomes to deliver an antisense oligonucleotide against androgen receptors for prostate cancer treatment. The overexpression and activation of androgen receptors have been related to the progression and resistance of prostate cancer. The iRGD-modified liposomes promoted an efficient lockdown of the androgen receptor gene, as demonstrated by in vitro assays. In vivo experiments using subcutaneous prostate cancer xenografts indicated that iRGD-modified liposomes significantly enhanced the antisense oligonucleotide against androgen receptor accumulation and reduced the androgen receptor expression in prostate tumors. Furthermore, iRGD-modified liposomes showed a higher reduction in tumor volume. Moreover, similar results were found using a xenografic model of bone metastasis. In addition, it was no observed antisense oligonucleotide against androgen receptor accumulation and toxicity in normal tissues [99]. 

TLyp-1 is a peptide that can specifically bind to the neuropilin receptor expressed in tumor cells. Yan et al. used its targeting property to produce targeted cationic liposomes for triple-negative breast cancer treatment by silencing the Slug gene with a miRNA, a gene associated with cell invasion, metastasis, and drug resistance. The cellular uptake assay indicated that tLyp-1-modified miRNA liposomes were internalized to a significantly greater extent than unmodified liposomes (about 12.7-folds) in MDA-MB-231 cells. In vivo assays have also identified the inhibition of *Slug* and *Blimp-1* expression in MDA-MB-231 cells, with a significant reduction in Slug and Blimp-1 protein levels. In addition, in vivo studies using a cancer-bearing animal model (BALB/c nude mice) demonstrated the greater antitumoral potential of tLyp-1-modified miRNA liposomes through the silence of *Slug* mRNA and Slug protein expression, which inhibited invasiveness and growth of triple-negative breast cancer cells [103]. 

Lin et al. developed cationic liposomes functionalized with GE-11 peptide to dual-target gemcitabine and HIF1α-siRNA (GE-GML/siRNA) for pancreatic cancer treatment through the specifically bind of GE-11 peptide to EGFR overexpressed. Gemcitabine is a potential antitumoral agent used as a first-line drug for pancreatic cancer treatment, whereas HIF1α-siRNA can downregulate the HIF1-α, a heterodimer related to tumor cells’ survival in hypoxia conditions. The results indicated that GE-11 increased the targeting specificity of liposomes to Panc-1 cells, and the siRNA was able to decrease the expression of the HIF1-α gene in vitro. Furthermore GE-GML/siRNA (IC_50_ = 0.42 µg/mL) showed significantly greater cytotoxicity in Panc-1 cells than free gemcitabine (IC_50_ = 8.56 µg/mL) and unmodified formulation (IC_50_ = 7.45 µg/mL). In addition, a synergistic effect was observed combining gemcitabine and siRNA. The functionalized liposomes reduced the tumor by twofold more when compared with unmodified liposomes, suggesting the potential of gemcitabine/HIF1α-siRNA-loaded GE-11-modified liposomes in pancreatic cancer treatment [102].

Shabana et al. investigated peptide-modified liposomes for delivering miRNA 603 (miR-603) to brain tumor cells. The miR-603 was used to downregulate insulin-like growth factor 1 signaling by suppressing the glioblastoma multiforme radiation resistance. To promote the specific targeting of this liposomal formulation to glioblastoma cells, the authors decorated this nanosystem with PR_b peptide that specifically targets the α5β1 integrin overexpressed in cancer cells. The functionalization of liposomes with PR_b peptide increased 22-fold the transfection of miR-603 and reduced insulin-like growth factor 1 (3.2-folds) and insulin-like growth factor 1 receptor mRNA (2.5-folds) expression in GBM-CCC-001 cells. The developed formulation was able to sensitize patient-derived glioblastoma stem-like cells to ionizing radiation, demonstrating that the decoration of liposomes with PR_b peptide is a potential system to transfect miR-603 to tumor cells and improve radiation sensitivity [16].

Yuan et al. have also developed peptide-modified liposomes for glioblastoma multiforme gene therapy using siRNA to silence Golgi phosphoprotein3 (GOLPH3) mRNA expression. The high levels of GOLPH3 in glioma patients are related to the low survival time because of its association with tumor cells proliferation. The angiopep-2 was the peptide chosen for active targeting the GOLPH3-siRNA-liposomes to glioblastoma cells because of its specific binding to LRP-1 receptors expressed in the blood–brain barrier and tumor cells. The angiopep-2-modified GOLPH3-siRNA-liposomes exhibited significantly silencing of the GOLPH3 mRNA (71.8%) and GOLPH3 protein (68.3%) expression in U87 cells. Furthermore, in vivo studies demonstrated the higher ability of angiopep-2-modified GOLPH3-siRNA-liposomes to accumulate in the brain and effectively inhibit glioma growth in comparison with free GOLPH3-siRNA, suggesting the potential of this liposomal formulation for glioblastoma treatment [104].

The studies mentioned demonstrated the advantages of using peptides for the active targeting of liposomes for gene therapy. However, it is important to highlight that the development of peptide-modified liposomes has some limitations and challenges that should be overcome. These limitations include: (I) the shift from the small scale to industry-scale production of functionalized liposomes maintaining their physicochemical features, (II) the development of proper methods to quantify the exact number of peptides on liposomes, (III) aggregation of liposomes due to high ligand density, and (IV) the nonspecifically binding of serum proteins to the peptides on liposomes surface [142]. In this context, further efforts are required to design peptides-modified liposomes and overcome these limitations, given that peptide functionalization has shown great potential for cancer gene therapy. 

### 4.3. Aptamers

**Figure 5:**
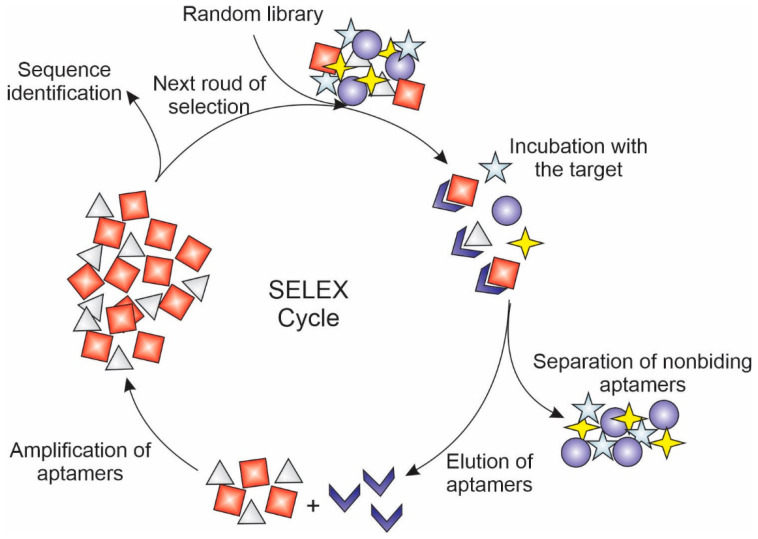
Schematic representation of sequential evolution of ligands by exponential enrichment (SELEX) used to select aptamers with high affinity and specificity by receptors overexpressed in tumor cells.

Aptamers are oligonucleotides or peptides that are usually synthesized by selecting them from a random sequence pool through a process called sequential evolution of ligands by exponential enrichment (SELEX) (Figure 5). According to their composition, aptamers can be classified as nucleic acid aptamer and peptide aptamer. The nucleic acid aptamer is a short strand of RNA or DNA oligonucleotides, while peptide aptamers consist of a short sequence of amino acids attached to a stable protein scaffold [150,151,152]. Their three-dimensional structure can bind with high affinity and specificity to receptors expressed in cancer cells, which make them great candidates for functionalizing liposomes for cancer gene therapy [153]. In addition, aptamers have a small size, low immunogenicity, relatively low production cost, and easiness of store, which make them great candidates for promoting the active targeting of NAs to cancer cells [154]. 

Aptamer AS1411 is one of the most described and used to target drugs or genes to tumor cells because of its specificity for nucleolin receptors, which is overexpressed in several cancer cell lines. The aptamer AS1411 competes with bcl-2 mRNA for biding to nucleolin, inducing apoptosis and reducing cell proliferation, mitogenesis, and angiogenesis of tumor cells [105,155]. The aptamer AS1411 was used to functionalize cationic liposome coloaded with polo-like kinase 1 siRNA and paclitaxel for breast cancer therapy. Gene therapy using siRNA targeting polo-like kinase 1 is a promising strategy, given that the overexpression of this protein in cancer cells is related to cell proliferation, metastasis, and angiogenesis. Targeted liposomes showed greater cytotoxicity and cellular uptake in MCF-7 cells, compared with the unmodified, with an IC_50_ value of 6.5-fold cytotoxic of the nonfunctionalized liposomes. The treatment also knocked down efficiently the polo-like kinase 1 mRNA (79%) in MCF-7 cells. The in vivo xenograft tumor model showed 62% inhibition of tumor growth, in addition to increased animal survival (~48 days), compared with the nonfunctionalized liposome (~26 days) [106]. 

Gharaibeh et al. also used the aptamer AS1411 for targeting siRNA-loaded cationic liposomes for breast cancer treatment. The authors used siRNA for silencing the Notch 1 protein, which is related to tumor cells proliferation. In vitro assays indicated a twofold greater uptake of functionalized liposomes, mainly located in the cytoplasm of MDA-MB-231 cells. Furthermore, targeted liposomes significantly reduced the expression of Notch 1 in MDA-MB-231 cells, while nonfunctionalized liposomes did not reduce their expression [15]. Li et al. used the same aptamer to functionalize cationic liposomes loaded with anti-BRAF siRNA for melanoma treatment. BRAF are mutant genes expressed in melanoma cells, being associated with tumor cell viability and transformation. In vitro assays demonstrated that targeted liposomes were internalized in A375 cells, which a consequent knockdown effect in the BRAF gene (~34.13%) and downregulation of BRAF protein expression. Moreover, targeted liposomes showed greater accumulation in tumor tissue than unmodified liposomes [105].

The RNA aptamer A10 can be specifically recognized and bind to prostate-specific membrane antigen highly expressed in prostate cancer cells. This aptamer was used by Zhen et al. to functionalize cationic liposomes loaded with CRISPR/Cas9 to prostate cancer cells. The CRISPR/Cas9 gRNA was used to target the prosurvival gene polo-like kinase 1. Functionalization with aptamer A10 provided a reduction of 63% in polo-like kinase 1 mRNA expression levels, with increased cellular uptake, cytotoxicity, and apoptosis in vitro. Furthermore, targeted liposomes showed a 2.6-fold reduction in xenographic tumor in vivo compared with nonfunctionalized liposomes [107].

EGFR aptamer can be specifically bound to EGFR, being used as a targeting ligand for promoting the active targeting of nanosystems to cancer cells with its overexpression. Dong et al. used EGFR aptamer for targeting cationic liposomes loaded with SATB1 siRNA to choriocarcinoma cells. The special AT-rich sequence binding protein 1 (SATB1) contributes to cancer growth and metastasis, which makes its downregulation by siRNA a promising strategy for cancer treatment. In vitro assay indicated a remarkable transfection of targeted liposomes, with a subsequent significant reduction in the SATB1 protein expression (~80%), while untargeted liposomes inhibited the expression by about 25%. In addition, targeted liposomes showed greater apoptosis induction (~2.3-fold higher compared with unmodified liposomes). The SATB1 suppression was also confirmed in in vivo studies using mice bearing JEC-3 choriocarcinoma, with ~80% and ~30% for targeted and untargeted liposomes, respectively. In addition, targeted liposomes showed greater tumor volume and weight inhibition (79.4% and 81.4%, respectively) than untargeted formulation (46.9% and 48.9%, respectively) [14].

The epithelial cell adhesion molecule (EpCAM) is a transmembrane glycoprotein overexpressed in solid tumors. Thus, the EpCAM aptamer can be conjugated with cationic liposomes to specifically promote target tumor cells. Zhao et al. developed EpCAM aptamer-modified cationic liposomes loaded with miR-139-5P (DiR-ANPs) for the therapy of colorectal cancer. The miR-139-5P was delivered by the liposomes for promoting re-expression in colorectal cancer cells, given that its downregulation in cancer cells is associated with cell migration and invasion. In vitro experiments indicated that targeted liposomes were more internalized in EpCAM-positive cells (HCT116 and HCT8) than EpCAM-negative cells (HeLa), with a subsequent endosomal escape of miR-139-5P. In vivo studies using an HCT8 tumor-bearing mouse model indicated that DiR-ANPs had a higher accumulation in the tumor tissue, which potentiated threefold tumor inhibition of colorectal cancer xenograft [108].

CD44 is another transmembrane glycoprotein highly expressed in solid tumors. It is related to tumor cell adhesion, migration, proliferation, and differentiation. Thus, Alshaer et al. developed negative liposomes functionalized with Apt1 CD44 aptamer (also named Apt1) and loaded with CD44 siRNA for active targeting and gene silence in breast cancer cells. Targeted liposomes showed a 1.8-fold increase in mRNA inhibition in MDA-MB-231 cells than unmodified liposomes. The mRNA silencing was confirmed in vivo in the orthotopic breast cancer model, resulting from the accumulation of functionalized liposomes in the tumor [50].

The aforementioned studies have reinforced the importance of aptamers for the specific targeting of nanocarriers to tumoral cells. To ensure the success of these targeted therapies, it is important to highlight that the conjugation of aptamers to liposomes can change their binding specificity and affinity by tumor cells. Thus, during aptamer-modified liposomes production, researchers should consider the influence of charge and density of aptamers on liposome surfaces to optimize aptamer binding ability [154].

### 4.4. Folate

**Figure 6:**
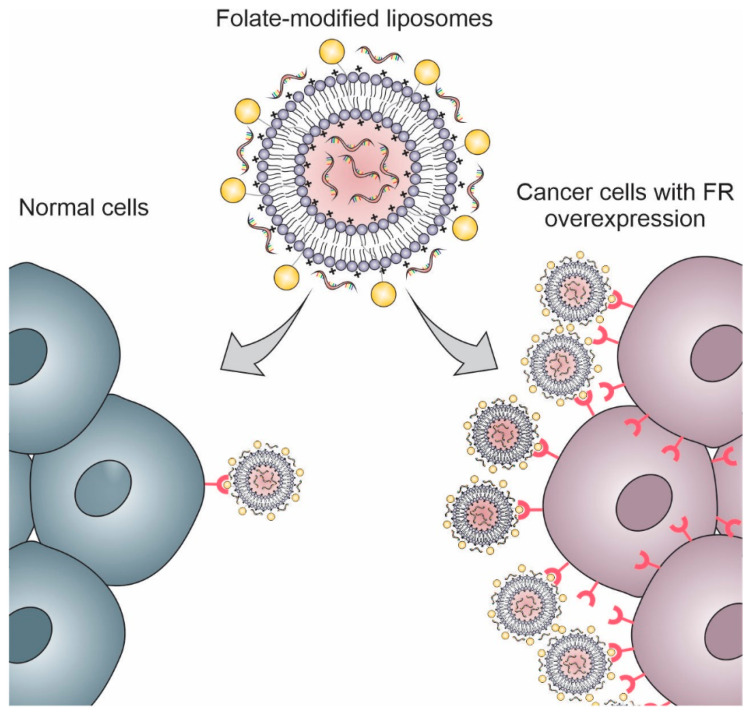
Schematic representation of folate receptors (FR) overexpression in cancer cells compared with normal cells.

Folate (vitamin B9) is a small molecule essential for the synthesis of purines and pyrimidines, and, as a consequence, it plays an important role in cell division and growth. The transport of this molecule into the cells occurs by four folate receptors isoforms (FRα, FRβ, FRγ, and FRδ). Among these isoforms, FRα has the most potential for active targeting, being overexpressed in cancers of the breast, ovary, lung, brain, prostate, colon, throat, and nose. It is reported that folate receptors are expressed 100–300 times more in tumoral cells than in normal cells (Figure 6). Besides the high affinity of folate by FRα, this molecule has the advantages of being inexpensive, nonimmunogenic, stable, easily manufactured, and easy to conjugate to nanosystems. In this way, the functionalization with folate is an interesting strategy for active targeting of the liposomes for cancer gene therapy [156,157,158].

Chen et al. modified a cationic liposomal formulation with folic acid for active targeting of the hypoxia-inducible factor-1α siRNA to malignant melanoma via folate receptors overexpressed. The hypoxia-inducible factor-1α siRNA was used to silence the hypoxia-inducible factor-1α gene expression, which is upregulated in tumoral cells and related to angiogenesis. The in vitro transfection assay using human melanoma cells (A375) indicated that folate-modified liposomes enhanced the siRNA transfection in comparison with unmodified liposomes, with mean fluorescence intensity similar to Lipofectamine™ 2000, a transfection reagent. Moreover, folate-modified liposomes showed higher in vitro antimelanoma activity and downregulation of hypoxia-inducible factor-1α protein than the unmodified formulation. Thus, the results demonstrated the potential of folate functionalization to improve the transfection of siRNA-loaded liposomes [159].

Folate-modified anti-MDR1 siRNA-loaded liposomes were developed for promoting the silence of the multidrug resistance (MDR1) gene upregulated in tumor cells. For this purpose, the authors synthesized a novel folate-containing lipoconjugate for active targeting siRNA-loaded liposomes in tumor cells. Folate-modified liposomes had 3–4-fold higher transfection efficiency in tumor cells than unmodified liposomes. In addition, in vivo studies indicated the high accumulation of folate-modified liposomes in tumors (around 15–18%), followed by an efficient downregulation of the MDR1 gene and p-glycoprotein expression (to 40% of the control group) in tumors, indicating the great potential of this formulation for cancer treatment [109]. Gladkikh et al. have also investigated folate-modified liposomes for MDR1-siRNA delivery. The author observed a greater accumulation of folate-modified liposomes in tumors composed of cells with high expression of folate receptors, confirming that the functionalization with folate is an interesting strategy for enhancing the targeting of nucleic acids [13].

Li et al. codelivered a siRNA against the Bmi1 gene with ursolic acid to tumor cells using folate-modified cationic liposomes. The upregulation of the Bmi1 gene has been related to the self-renewal and malignancy of stem cells. Thus, the downregulation of this gene by siRNA combined with the antitumoral activity of ursolic acid is a strategy to inhibit tumor growth. In vitro experiments indicated that folate-modified liposomes codelivering ursolic acid and Mni1 siRNA (FA-UA/siRNA-L) have significantly higher cellular uptake and cytotoxicity effects when compared with the unmodified liposomes, with a synergic antitumor effect. In addition, the Western blotting assay demonstrated significantly lower Bmi1 expression when FA-UA/siRNA-L was compared with the unmodified formulation. In vivo studies using the subcutaneous tumor-bearing balb/c mice model reinforced the antitumoral results observed in vitro, demonstrating the synergistic antitumoral effect of FA-UA/siRNA-L [110].

Overexpression of folate receptors in tumor cells was also used by Yang et al. as a strategy to deliver folate-modified cationic liposomes containing the exogenous pigment epithelium-derived factor (PEDF) gene to cervical cancer cells. PEDF is a glycoprotein that provides antitumoral properties of apoptosis, differentiation, anti-proliferation, and anti-angiogenesis. The PEDF plasmid was complexed with targeted liposomes to overcome the low expression of PEDF in cervical cancer. In vitro assays demonstrated that Folate-modified PEDF-loaded liposomes (FLP) transfected HeLa cells significantly more than unmodified formulation, in addition, to inhibiting cell growth, adhesion, invasion, and migration. Moreover, the PEDF gene was upregulated in tumor tissues and serum, leading to angiogenesis suppression, cell proliferation inhibition, and cell apoptosis induction in vivo. The result demonstrated the importance of PEDF upregulation and the use of folate as a targeting ligand on liposomes for specific transfection of the selected gene [111].

Liang et al. developed folate-modified liposomes loading recombinant interleukin-15 (IL15) plasmid (F-PLP/pIL15) for specific targeting of colon cancer cells via folate receptors. IL-15 is a cytokine used for cancer immunotherapy due to its ability to modulate tumors. However, its systemic administration can cause several side effects, including hypotension, thrombocytopenia, and liver injury. In vitro assay indicated that F-PLP/pIL15 significantly increased IL15 secretion by colon cancer cells (CT26). The intraperitoneal administration of F-PLP/pIL15 in mice significantly inhibited the tumor growth, reducing the tumor nodules (88 to 43 nodules) and tumor weight (5.43 g to 1.88 g), with no detectable toxicity. The in vivo cell proliferation levels were assessed by Ki_67_ staining. F-PLP/pIL15 showed significantly fewer proliferation cells in tumor tissues (16%) than in unmodified formulation (37%) or the control group (65%). These results suggested that folate is a potential target ligand for promoting specific tumor damage in colon cancer therapy [160]. 

## 5. Conclusions and Perspectives

Gene therapy has become a promising strategy for the treatment of cancer. For this purpose, the development of a gene vector able to specifically deliver and transfect nucleic acid to tumor cells plays an important role in the success of gene therapy. In this context, functionalized liposomes have demonstrated great ability to transfect tumor cells due to their recognition with high specificity and affinity by receptors overexpressed in tumor cells. The design of these nanosystems has used different lipids to produce liposomes (anionic, cationic, and neutral). Among them, lipids with cationic headgroup have demonstrated better features for gene delivery, including the easy complexation between lipid and genes by electrostatic interaction, great interaction with cell membrane through electrostatic interaction, and ability to escape from the endosomes by their interaction and fusion with the endosome membrane. In addition, the choice of the targeting ligand can influence the ability of liposomes to transfect cells. During the choice of the ligand (e.g., antibodies, antibodies fragment, peptides, aptamers, folic acid, carbohydrates), it is important to evaluate the level of its target receptor, the orientation of the ligand in de liposomes surface, and the ligand density to guarantee the proper bind to a target receptor. In vitro and in vivo studies using different targeting ligands have suggested that the functionalization of liposomes can enhance the delivery of genes into tumor cells, with a consequent improvement in their antitumoral activity when compared with unmodified liposomes. However, to the best of our knowledge, to date, few clinical trials have been performed using liposomes loaded with nucleic acids for cancer therapy. In addition, clinical trials using functionalized liposomes have not yet been performed, demonstrating the need for further studies for a greater understanding of the targeting ability and antitumoral potential of functionalized liposomes loaded with nucleic acids.

## Figures and Tables

**Table 1:** Clinical trials for cancer gene therapy.

Disease	Vector/Gene	Phase	Status	Company	Identifier
Ovarian Cancer and Peritoneal Cavity Cancer	Ad5CMV-p53 gene	I	Completed	University of Texas Southwestern Medical Center	NCT00003450
Pancreatic cancer	Rexin-gene	I	Completed	Epeius Biotechnologies	NCT00121745
Prostate cancer	Ad5-yCD/mutTKSR39rep-hIL12	I	Unknown	Henry Ford Health System Detroit	NCT02555397
Non-Small Cell Lung Cancer	AdV-IL-12	I	Incomplete	Houston Methodist Cancer Center	NCT04911166
Triple-Negative Breast Cancer	AdV-IL-12	II	Incomplete	Houston Methodist Cancer Center	NCT04095689
Breast Cancer	Ad5CMV-p53 gene	I	Completed	Fox Chase Cancer Center Philadelphia	NCT00004038
Ovarian Cancer and Primary Peritoneal Cancer	Ad5CMV-p53	I	Completed	Simmons Cancer Center—Dallas	NCT00003450
Prostate Cancer	Ad5-CMV-NIS	I	Completed	Mayo Clinic Rochester	NCT00788307

**Table 2:** Lipids commonly used for gene transfer.

Lipid	Abbreviation	Polar Domain	Nonpolar Domain	Feature
*N*-[1-(2,3-Dioleyloxy)propyl]*N*,*N*,*N*-trimethylammonium chloride	DOTMA	Quaternary ammonium	Unsaturated aliphatic	Cationic lipid
1,2-Dioleoyloxy-3-trimethylammonium-propane	DOTAP	Quaternary ammonium	Unsaturated aliphatic	Cationic lipid
Dioctadecylamidoglycylspermine	DOGS	Polyamine	Aliphatic	Cationic lipid
Cetyltrimethylammonium bromide	CTAB	Quaternary ammonium	Single-tail aliphatic	Cationic lipid
2,3-Dioleyloxy-*N*-[2(sperminecarboxamido)-ethyl]-*N*,*N*-dimethyl-1-propanaminium trifluoroacetate	DOSPA	Polyamine	Unsaturated aliphatic	Cationic lipid
1,2-Dioleyl-3-trimethylammonium-propane	DOPA	Quaternary ammonium	Unsaturated aliphatic	Cationic lipid
Dimyristooxypropyl dimethyl hydroxyethyl ammonium bromide	DMRIE	Quaternary ammonium	Aliphatic	Cationic lipid
Dimethyldioctadecylammonium bromide	DDAB	Quaternary ammonium	Aliphatic	Cationic lipid
1,2-Distearyloxy-*N*,*N*-dimethyl-3-aminopropane	DSDMA	Secondary amine	Aliphatic	Cationic lipid
1,2-Dimyristoyl-trimethylammoniumpropane	DMTAP	Quaternary ammonium	Aliphatic	Cationic lipid
1,2-Distearoyl-sn-glycero-3-ethylphosphocholine	DSEPC	Quaternary ammonium	Aliphatic	Cationic lipid
*N*-Palmitoyl d-erythro-sphingosyl carbamoyl-spermine	CCS	Spermine	Unsaturated aliphatic	Cationic lipid
1,3-Dioleoxy-2-(6-carboxy-spermyl)-propylamide	DOSPER	Polyamine	Unsaturated aliphatic	Cationic lipid
(1,2-dioleoyl-3-dimethyl-hydroxyethyl ammonium bromide)	DORIE	Quaternary ammonium	Unsaturated aliphatic	Cationic lipid
(1,2-dioleoyloxypropyl-3-dimethyl-hydrox yethyl ammonium chloride)	DORI	Quaternary ammonium	Unsaturated aliphatic	Cationic lipid
*N*,*N*-dioleyl-*N*,*N*-dimethylammonium Chloride	DODAC	Quaternary ammonium	Aliphatic	Cationic lipid
Bis-guanidium-tren-cholesterol	BGTC	Guanidinium-spermidine-	Steroid-based	Cationic lipid
3β-[*N*-(*N*′,*N*′-Dimethylaminoethane)-carbamoyl]cholesterol	DC-Chol	Tertiary amine	Steroid-based	Cationic lipid
Octadecenolyoxy[ethyl-2-heptadecenyl-3 hydroxyethyl] imidazolinium chloride	DOTIM	Heterocycle (imidazole)	Unsaturated aliphatic	Cationic lipid
1,2-dioleoyl-sn-glycerol-3-ethylphosphocholine	DOEPC	Ethylphosphocholine	Aliphatic	Cationic lipid
*O*,*O*′-Dimyristyl-*N*-lysyl aspartate	DMKE	Primary amine	Aliphatic	Cationic lipid
*O*,*O*′-dimyristyl *N*-lysylaspartate	DMKD	Primary amine	Aliphatic	Cationic lipid
*N*-t-Butyl-*N*0-tetradecyl-3-tetradecylaminopropionamidine	diC14-amidine	Imine group	Aliphatic	Cationic lipid
*N*-(4-carboxybenzyl)-*N*,*N*-dimethyl-2,3-bis(oleoyloxy)propan-1-aminium	DOBAQ	Quaternary ammonium	Unsaturated aliphatic	Cationic lipid
1,2-dioleyloxy-3-dimethylaminopropane	DODMA	Tertiary amine	Unsaturated aliphatic	Cationic lipid
6-Lauroxyhexyl ornithinate	LHON	Ornithine	Single-tail aliphatic	Cationic lipid
1,2-Dioleoyl-sn-glycero-3-phosphatidylcholine	DOPC	-	Phosphatidylcholine	Helper lipid
1,2-Dioleoyl-sn-glycero-3-phosphatidylethanolamine	DOPE	-	Phosphatidylcholine	Helper lipid
Cholesterol	CHOL	-	Steroid	Helper lipid

**Table 3:** Active clinical trials of lipoplex-based delivery systems containing nucleic acids for cancer treatment.

Lipid	Gene/Drug	Disease	Administration Route	Phase (Start Year)	Sponsors	Identifier
n.r.	mRNA encoding human OX40L	Advanced/metastatic solid tumors or lymphoma	Intratumoral	I/II recruiting (2017)	ModernaTX, Inc. (Cambridge, MA, USA)	NCT03323398
Lipo-MERIT	NY-ESO-1, MAGE-A3, and TPPE RNA	Melanoma	Intravenous	I recruiting (2015)	BioNTech SE (Mainz, Germany)	NCT02410733
DOTAP:Chol	Pbi-shRNA™ EWS/FLI1 Type 1	Ewing’s sarcoma.	Intravenous	I recruiting (2016)	Gradalis, Inc. (New York, NY, USA)	NCT02736565
DOPC	EphA2 siRNA	Advanced Malignant Solid Neoplasm	Intravenous	I active (2012)	M.D. Anderson Cancer Center (Houston, TX, USA)	NCT01591356
DOTAP:Chol	TUSC2	Lung Cancer	Intravenous	I/II active (2011)	Genprex, Inc. (Houston, TX, USA)	NCT01455389
DOTAP:DOPE	SGT-53	Recurrent/refractory solid tumors in children	Intravenous	I active (2015)	SynerGene Therapeutics, Inc. (Houston, TX, USA)	NCT02354547
DOTAP:DOPE	SGT-53	Metastatic pancreatic cancer	Intravenous	II recruiting (2015)	SynerGene Therapeutics, Inc. (Houston, TX, USA)	NCT02340117

NY-ESO-1—New York-ESO 1; MAGE-A3—tyrosinase, Melanoma-associated antigen A3; TPTE—Transmembrane phosphatase with tensin homology; TUSC2—Tumor suppressor candidate 2; SGT-53—a complex of cationic liposome encapsulating a normal human wild type p53 cDNA sequence in a plasmid backbone; n.r.—not reported.
